# Upper Gastrointestinal Cancer Audit: A Retrospective Analysis of Patient Outcomes in North Wales

**DOI:** 10.7759/cureus.111600

**Published:** 2026-06-27

**Authors:** Sulochan Lohani, Divneet Chadha, Shanti Gautam, Sarala Pokharel, Muhammad Khan, Oladapo Adesua, Ravi Pudasaini, Mohsin Khan, Angel Garcia

**Affiliations:** 1 General Internal Medicine, Betsi Cadwaladr University Health Board, Bodelwyddan, GBR; 2 General Practice, Betsi Cadwaladr University Health Board, Wrexham, GBR; 3 Ear, Nose, and Throat, Betsi Cadwaladr University Health Board, Bodelwyddan, GBR; 4 General Medicine, Betsi Cadwaladr University Health Board, Bodelwyddan, GBR; 5 Emergency Medicine, Betsi Cadwaladr University Health Board, Bodelwyddan, GBR; 6 Oncology, Betsi Cadwaladr University Health Board, Bodelwyddan, GBR

**Keywords:** flot chemotherapy, general surgery complication, gi oncology, north wales, retrospective studies, upper gi cancer

## Abstract

Background: Upper gastrointestinal (UGI) cancers, including esophageal, gastroesophageal junction (GOJ), and gastric adenocarcinomas, remain clinically important malignancies associated with late presentation, multimodal treatment requirements, and treatment-related morbidity. Real-world institutional audits can help describe outcomes outside clinical trial settings and identify areas for service improvement.

Objective: The primary objective was to describe crude-documented two-year progression-free status among patients undergoing active treatment for UGI adenocarcinoma at Betsi Cadwaladr University Health Board in North Wales, United Kingdom. The secondary objectives were to describe baseline and tumor characteristics, treatment patterns, postoperative complications, and 30-day postoperative mortality.

Methods: A retrospective audit was conducted, including 78 patients with UGI cancers between January 2019 and May 2024 who received active oncological treatment. Data were extracted from institutional oncology records, multidisciplinary team documentation, pathology reports, and electronic medical records. Two-year outcome status was reported using the full audit cohort (n = 78), whereas surgical outcomes, resection margin status, pathological response, postoperative complications, and 30-day postoperative mortality were reported using the surgically treated cohort (n = 56). Because event timing, censoring, and follow-up intervals were not consistently documented, two-year progression-free status was reported as a crude descriptive outcome proportion rather than a formal Kaplan-Meier survival estimate.

Results: Seventy-eight patients were included; the median age was 74 years, and 63 (80.8%) were male patients. Tumor sites were gastric in 50 (64.1%), esophageal in 26 (33.3%), and GOJ in two (2.6%). All 78 patients received neoadjuvant fluorouracil, leucovorin, oxaliplatin, and docetaxel chemotherapy. Surgical resection was performed in 56 out of 78 patients (71.8%). Among surgically treated patients, R0 resection was documented in 27 (48.2%), R1 resection in 11 (19.6%), and margin status was not explicitly stated in 18 (32.1%). Thirty-day postoperative mortality was one (1.8%). Retrospectively documented postoperative complications included sepsis in two (3.6%), pneumonia in one (1.8%), and anastomotic leak in one (1.8%). These complications were retrospectively classified based on available documentation; however, formal Clavien-Dindo grading could not be applied consistently due to incomplete retrospective severity documentation. Of 78 patients, crude-documented two-year progression-free status was observed in 30 patients (38.5%), 32 (41.0%) had documented progression or death within two years, and 16 (20.5%) were not evaluable due to incomplete follow-up documentation.

Conclusion: This retrospective institutional audit provides descriptive real-world data on treatment patterns and documented outcomes among patients with UGI adenocarcinoma managed within a National Health Service health board. The findings should be interpreted as descriptive audit data rather than evidence of treatment efficacy or equivalence with clinical trial outcomes. Prospective data collection with standardized recording of performance status, comorbidity, treatment completion, dose modifications, surgical procedure type, complication grading, follow-up duration, and time-to-event outcomes would strengthen future evaluation of this cohort.

## Introduction

Upper gastrointestinal (UGI) cancers remain a leading cause of cancer-related morbidity and mortality worldwide [1]. According to Global Cancer Observatory estimates, gastric and esophageal cancers collectively account for more than 1.6 million new cancer diagnoses and almost 1.3 million cancer-related deaths annually worldwide [1]. Esophageal and gastric cancers are particularly aggressive malignancies, often characterized by late-stage diagnosis, rapid progression, and poor overall survival.

The clinical presentation of UGI cancers is frequently nonspecific in early stages, resulting in delayed diagnosis and advanced disease at presentation. This contributes significantly to poor prognosis and limits the effectiveness of curative interventions [2].

Over the past two decades, management strategies have evolved toward a multimodal approach combining chemotherapy and surgery. Landmark trials have demonstrated that perioperative chemotherapy improves survival compared to surgery alone in patients with resectable disease [3]. The evolution of perioperative chemotherapy from the Medical Research Council Adjuvant Gastric Infusional Chemotherapy regimen to the fluorouracil, leucovorin, oxaliplatin, and docetaxel (FLOT4) protocol has significantly influenced modern management of UGI cancers, with FLOT demonstrating improved survival outcomes in resectable disease [3,4]. More recently, regimens such as FLOT have shown superior outcomes and are now widely adopted in clinical practice [4].

Despite these advances, treatment-related morbidity remains a significant concern. Postoperative complications, including pneumonia, anastomotic leaks, and surgical site infection, are common and have been shown to adversely affect both short-term recovery and long-term oncological outcomes [5].

Clinical trial outcomes are not always directly generalizable to routine practice because trial cohorts may differ from real-world patients in age, comorbidity, performance status, tumor stage distribution, treatment completion, operative fitness, and follow-up methodology. Retrospective institutional audits can, therefore, provide useful descriptive information about local treatment delivery, postoperative outcomes, and documentation gaps. This audit was undertaken to describe treatment patterns and outcomes among patients with UGI adenocarcinoma managed within Betsi Cadwaladr University Health Board (BCUHB).

## Materials and methods

Study design and setting

This study was conducted as a retrospective clinical audit at BCUHB, National Health Service (NHS) Wales, United Kingdom (Audit Registration ID: 2263; approval date: 09/06/2025). The audit evaluated patients diagnosed with UGI cancers between January 2019 and May 2024 who received active oncological treatment (chemotherapy and/or surgical intervention).

Study population

A total of 78 patients with UGI cancers were included in the audit. Patients were identified retrospectively through institutional oncology databases, multidisciplinary team (MDT) records, pathology reports, and electronic medical records. Eligible patients were adults aged 18 years or older with histologically confirmed esophageal, gastroesophageal junction (GOJ), or gastric adenocarcinoma who received active oncological treatment, including chemotherapy and/or surgical intervention. Patients with nonadenocarcinoma histology, insufficient key clinical documentation, or purely palliative management without active oncological treatment were excluded. The full cohort of 78 patients forms the denominator for progression-free survival (PFS) and overall treatment pattern analyses. Postoperative outcomes (complications, 30-day mortality) are reported using the surgically treated cohort (n = 56) as the denominator, as these outcomes apply only to patients who underwent resection.

Inclusion criteria

Patients were included in the study if they were aged 18 years or older; had histologically confirmed esophageal, GOJ, or gastric adenocarcinoma; and underwent active oncological treatment, including chemotherapy and/or surgical intervention. Selection bias may have been introduced by exclusion of patients managed with purely palliative intent, potentially limiting generalizability and influencing survival outcomes.

Exclusion criteria

Patients were excluded if essential diagnostic or treatment data were insufficient to confirm eligibility, had nonadenocarcinoma histology, or were managed with purely palliative intent without receiving active oncological treatment. Because only patients receiving active treatment were included, the findings may not generalize to the broader population of patients with UGI cancers.

Data collection

Variables extracted included age, sex, tumor site, histology and differentiation, human epidermal growth factor receptor 2 status, clinical T, N, and M stage, chemotherapy regimen, documented number of perioperative chemotherapy cycles, surgery status, resection margin status, pathological tumor regression grade where available, documented treatment-related toxicities, postoperative complications, 30-day postoperative mortality, and crude two-year progression-free status. Several clinically important variables were not consistently available in structured retrospective records, including Eastern Cooperative Oncology Group (ECOG) performance status, comorbidity burden, overall clinical stage grouping, treatment intent as a structured variable, chemotherapy dose reductions, surgical procedure type, Clavien-Dindo or Common Terminology Criteria for Adverse Event (CTCAE) complication grade, median follow-up duration, censoring status, and precise timing of progression.

Outcome measures

The primary outcome was crude documented two-year progression-free status. Patients were classified as progression-free at two years if available documentation showed no evidence of disease progression or death within two years of diagnosis. Patients with documented progression or death within two years were classified as not progression-free. Patients without sufficient documentation were classified as not evaluable or not recorded for this endpoint. Denominators were stated for each outcome. Baseline characteristics, tumor variables, chemotherapy exposure, and crude two-year progression-free status were calculated using the full audit cohort (n = 78). Surgical outcomes, resection margins, pathological tumor regression, postoperative complications, and 30-day postoperative mortality were calculated among surgically treated patients (n = 56). Documented chemotherapy cycle distributions were reported using the full audit cohort, while also acknowledging incomplete cycle documentation.

Confounding factors

Potential confounding factors included patient age, comorbidities, tumor stage at diagnosis, and treatment tolerance. Due to the retrospective nature of the study, adjustment for confounders was limited.

Statistical analysis

Descriptive statistical analysis was performed. Continuous variables were expressed as medians with ranges, and categorical variables were presented as frequencies and percentages. No inferential statistical tests were performed because of the audit design, small sample size, missing variables, and the absence of standardized time-to-event data. Formal Kaplan-Meier analysis, median PFS, median follow-up, and censoring analysis were not performed because follow-up timing and event dates were not consistently documented.

Ethical considerations

This study was conducted as a clinical audit. Approval was obtained from the institutional clinical audit department at BCUHB. As this was a retrospective audit of anonymized data, formal approval from the ethics committee (Institutional Review Board/Independent Ethics Committee) was not required. The audit was approved through institutional clinical governance procedures in accordance with local audit policy requirements (Audit Registration ID: 2263; approval date: 09/06/2025).

## Results

Patient demographics

**Figure 1:**
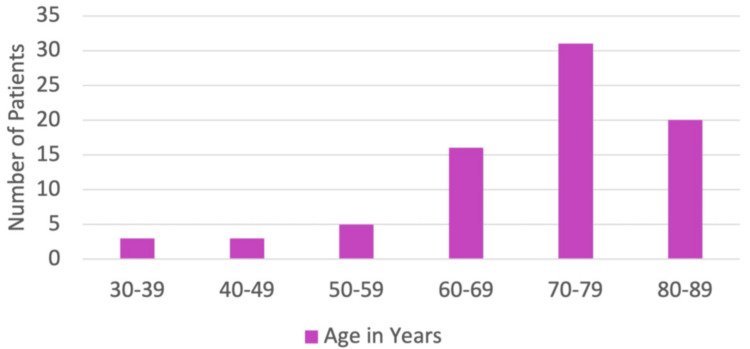
Age distribution of patients diagnosed with upper gastrointestinal cancers Age distribution of analyzed patients (n = 78). Values represent absolute patient numbers

A total of 78 patients with UGI cancer who received active treatment were included in the audit. The median age was 74 years (range: 31-87 years), reflecting an elderly population. Of these, 63 (80.8%) were male patients, consistent with known epidemiological patterns of UGI cancers [1]. All 78 patients (100%) received neoadjuvant chemotherapy with the FLOT regimen. Documentation of the number of preoperative and postoperative cycles showed variation based on available retrospective records; however, treatment completion rates, dose reductions, and delays were not consistently documented across the full cohort and should be interpreted descriptively only. Of the 78 patients, surgical resection was performed in 56 (71.8%), seven (9.0%) were deemed inoperable, and 15 (19.2%) had unknown/not recorded surgical status. Detailed surgical procedure types (e.g., esophagectomy vs. gastrectomy, open vs. minimally invasive) were not consistently available in the retrospective records. The age distribution of the study population is illustrated in Figure 1.

Tumor characteristics

Of 78 patients, gastric carcinoma was the most frequent tumor site (50, 64.1%), followed by esophageal carcinoma (26, 33.3%) and GOJ carcinoma (2, 2.6%). Poorly differentiated adenocarcinoma was documented in 32 (41.0%), moderately differentiated adenocarcinoma in 29 (37.2%), and well differentiated adenocarcinoma in one (1.3%); differentiation was not specified in 16 (20.5%). Poorly differentiated adenocarcinoma represented the predominant histological subtype within the cohort, indicating aggressive tumor biology. Forty-two patients (53.8%) presented with T3 stage disease, suggesting advanced local tumor invasion at diagnosis.

Treatment patterns

**Figure 2:**
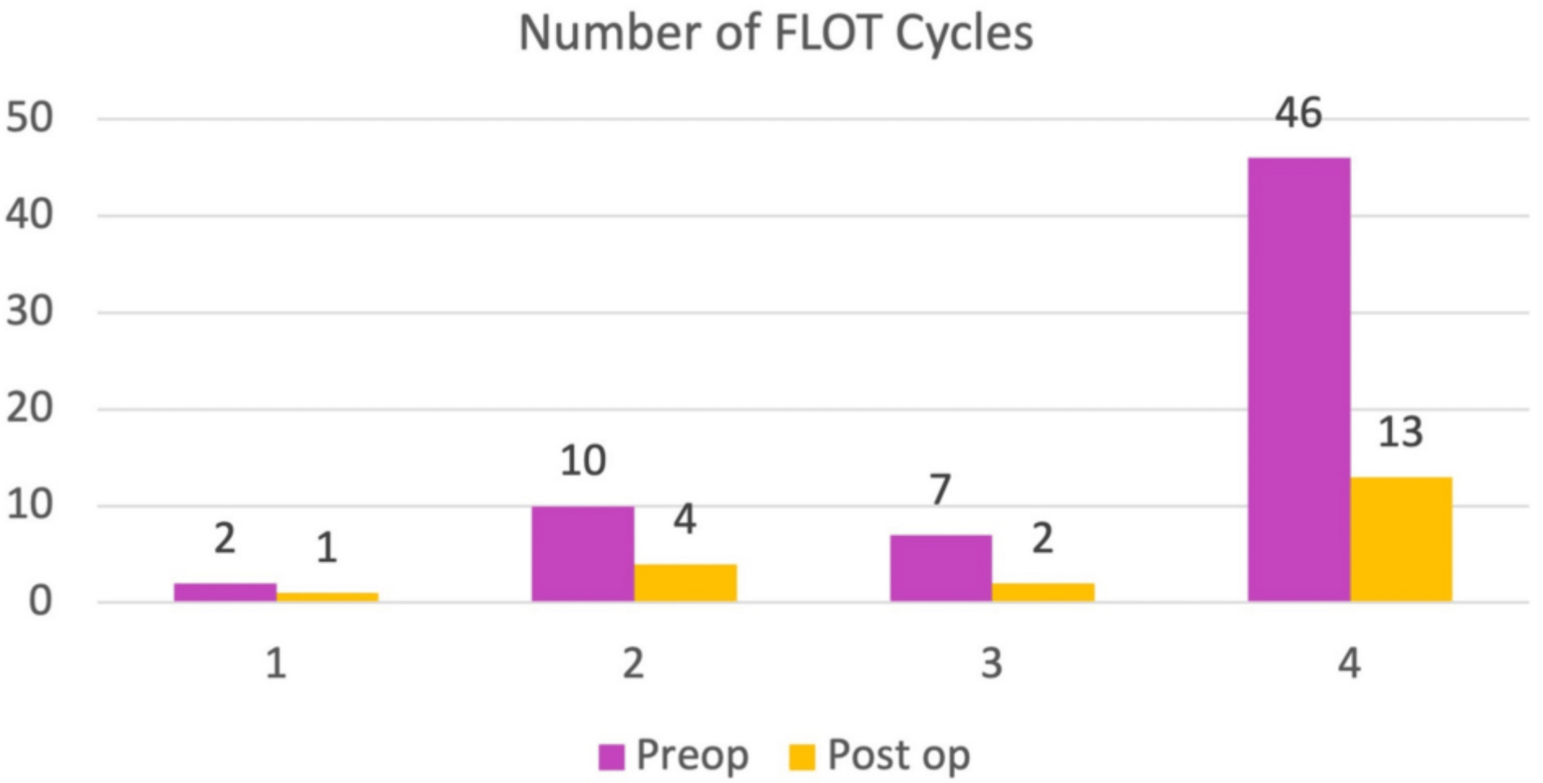
Documented distribution of perioperative chemotherapy cycles using the FLOT regimen Cycle counts are presented descriptively based on available retrospective documentation Preop: preoperative chemotherapy; Postop: postoperative chemotherapy; FLOT: fluorouracil, leucovorin, oxaliplatin, and docetaxel

All patients received neoadjuvant chemotherapy using the FLOT regimen. Of 78 patients, surgery was performed or attempted in 56 patients (71.8%), seven patients (9.0%) were recorded as not undergoing surgery due to inoperability, and the surgery status was unknown or not recorded for 15 (19.2%). Detailed surgical procedure type was not consistently available in the retrospective dataset. Available chemotherapy cycle documentation showed variation in the number of recorded preoperative and postoperative FLOT cycles. However, treatment completion and dose-reduction data were not consistently documented across the full cohort and should, therefore, be interpreted descriptively. The distribution of perioperative chemotherapy cycles using the FLOT regimen is presented in Figure 2.

Surgical outcomes and complications

Surgical resection was performed in 56 of 78 patients (71.8%). Postoperative events were reported using the surgically treated cohort (n = 56) as the denominator. Retrospectively documented postoperative complications included sepsis in two (3.6%), pneumonia in one (1.8%), and anastomotic leak in one (1.8%). These events were nonmutually exclusive. The retrospective dataset did not allow reliable calculation of a patient-level overall complication incidence, and formal Clavien-Dindo grading could not be consistently applied. Chemotherapy-related toxicities were also nonmutually exclusive and were reported descriptively using the full cohort denominator (n = 78).

Survival outcomes

Crude-documented two-year progression-free status was observed in 30 of 78 patients (38.5%). Thirty-two patients (41.0%) had documented progression or death within two years, and 16 of 78 (20.5%) were not evaluable or not recorded for this endpoint. Because exact event times, censoring status, and follow-up intervals were not consistently available, Kaplan-Meier analysis, median PFS, and median follow-up could not be reliably calculated. The reported figure should, therefore, be interpreted as a crude descriptive two-year outcome proportion, not a formal survival estimate.

Summary of clinical outcomes

A summary of demographics, tumor site, histology, and staging is presented in Table 1.

**Table 1:** Demographics, tumor site, histology, and staging (n = 78) Data were collected retrospectively from available clinical records; therefore, several variables had incomplete or inconsistently documented information. Overall clinical stage, ECOG performance status, comorbidity burden, detailed surgical procedure type, and standardized follow-up duration were not consistently available in retrospective records and could not be reliably analyzed. Percentages rounded to one decimal place UGI: upper gastrointestinal; GOJ: gastroesophageal junction; HER2: human epidermal growth factor receptor 2; ECOG: Eastern Cooperative Oncology Group

Variable/category	Value, n (%)
Demographics	
Total patients, n (%)	78 (100%)
Age (years), median (range)	74 (31-87)
Sex, n (%)	
Male	63 (80.8%)
Female	15 (19.2%)
Tumor site (UGI), n (%)	
GOJ carcinoma	2 (2.6%)
Carcinoma of esophagus	26 (33.3%)
Gastric carcinoma	50 (64.1%)
Histology and differentiation, n (%)	
Poorly differentiated adenocarcinoma	32 (41.0%)
Moderately differentiated adenocarcinoma	29 (37.2%)
Well differentiated adenocarcinoma	1 (1.3%)
Not specified	16 (20.5%)
HER2 status, n (%)	
Negative	40 (51.3%)
Positive	9 (11.5%)
Not reported	29 (37.2%)
Pretreatment clinical staging (cT), n (%)	
T1	3 (3.8%)
T2	20 (25.6%)
T3 (most common)	42 (53.8%)
T4 (incl. T4a)	6 (7.7%)
Missing/Tx	7 (9.0%)
Pretreatment clinical staging (cM), n (%)	
M0	67 (85.9%)
M1	2 (2.6%)
Missing/Mx	9 (11.5%)
Pretreatment clinical staging (cN), n (%)	
N0	28 (35.9%)
N1	36 (46.2%)
Missing/Nx	14 (17.9%)

A summary of treatment, surgery, resection margins, outcomes, and complications is presented in Table 2.

**Table 2:** Treatment, surgery, resection margins, outcomes, and complications Complications are listed separately and may overlap; no patient-level total or CTCAE/Clavien-Dindo grading was available from retrospective records. Data were collected retrospectively from available clinical records; therefore, several variables had incomplete or inconsistently documented information. Detailed surgical procedure type and structured complication grading were not consistently available in the retrospective dataset and could not be reliably analyzed. PFS is reported as crude binary status at two years (no Kaplan-Meier or time-to-event analysis performed) FLOT: fluorouracil, leucovorin, oxaliplatin, and docetaxel; TRG: tumor regression grade; PFS: progression-free survival; GI: gastrointestinal; CTCAE: Common Terminology Criteria for Adverse Event

Variable/category	Value, n (%)
Neoadjuvant chemotherapy	
Neoadjuvant FLOT chemotherapy	78 (100.0%)
Surgery performed	
Yes (resection attempted)	56 (71.8%)
No (inoperable)	7 (9.0%)
Unknown/not recorded	15 (19.2%)
Resection margins (surgery performed, n = 56)	
R0 (complete resection, negative margins)	27 (48.2%)
R1 (microscopic positive margin)	11 (19.6%)
R status not explicitly stated	18 (32.1%)
Pathological response (Becker TRG)	
TRG 1a: complete regression (0% residual)	3 (5.4%)
TRG 1b: <10% residual tumor	11 (19.6%)
TRG 2: 10%-50% residual tumor	7 (12.5%)
TRG 3: >50% residual/no regression	16 (28.6%)
Other/unclear TRG	19 (33.9%)
Postoperative mortality	
30-day postoperative mortality	1 (1.8%)
Crude documented 2-year PFS status	
Yes (progression-free)	30 (38.5%)
No (progressed or died)	32 (41.0%)
Not evaluable or not recorded	16 (20.5%)
Chemotherapy-related complications (no systematic grading; nonmutually exclusive)	
GI toxicity (diarrhea/vomiting/nausea)	17 (21.8%)
Fatigue	12 (15.4%)
Peripheral neuropathy	10 (12.8%)
Postoperative/surgical complications (no systematic grading)	
Sepsis	2 (3.6%)
Pneumonia	1 (1.8%)
Anastomotic leak	1 (1.8%)

## Discussion

This study provides important real-world insight into the outcomes of patients with UGI cancers managed in an NHS setting. The cohort was elderly and predominantly male, and included a high proportion of gastric tumors and cT3 disease. All included patients received neoadjuvant FLOT chemotherapy, and 56 of 78 patients underwent or had attempted surgical resection. Of 78 patients, crude-documented two-year progression-free status was observed in 30 patients (38.5%), 32 patients had documented progression or death within two years, and 16 were not evaluable or not recorded for this endpoint. The findings highlight the challenges associated with advanced disease presentation, treatment-related morbidity, and survival outcomes.

Advanced disease at presentation

The predominance of T3-stage tumors (53.8%) and poorly differentiated histology (41.0%) reflects late diagnosis, consistent with global epidemiological trends [1]. Early-stage UGI cancers are often asymptomatic, contributing to delayed diagnosis and poor prognosis [2]. This underscores the need for improved early detection strategies.

Effectiveness of multimodal therapy and real-world context

Crude-documented two-year PFS status was observed in 30 of 78 patients (38.5%). Although this proportion may be viewed in the context of published perioperative chemotherapy studies, direct comparisons with clinical trials such as FLOT4 should be interpreted with caution because of differences in study design, patient selection, tumor stage distribution, treatment completion, surgery rates, and follow-up methodology. The present study was a retrospective descriptive audit and was not designed to establish treatment efficacy or equivalence with trial outcomes [4]. The findings provide valuable descriptive, real-world data on treatment patterns and observed outcomes in routine NHS practice, rather than on efficacy benchmarks.

Postoperative complications

Specific postoperative complications were documented in the surgically treated cohort (n = 56), including sepsis (3.6%), pneumonia (1.8%), and anastomotic leak (1.8%). Formal Clavien-Dindo grading could not be consistently applied due to incomplete retrospective documentation, which limits the interpretation of complication severity. These findings should therefore be interpreted as retrospectively recorded postoperative events rather than a standardized prospective complication assessment. Postoperative complications are known to negatively impact survival by delaying or preventing further treatment [5]. Additionally, systemic inflammatory responses may contribute to tumor progression [6].

Importance of perioperative optimization

The observed outcomes highlight the need for improved perioperative care in this high-risk population. Potential strategies include prehabilitation, which focuses on improving physical fitness and nutritional status prior to surgery [7], and Enhanced Recovery After Surgery (ERAS) protocols, which aim to reduce complications and shorten hospital stay [8]. The implementation of these strategies may significantly improve patient outcomes.

Role of multidisciplinary care

MDT involvement is essential in optimizing treatment decisions and improving outcomes [9]. Personalized treatment planning based on patient fitness and tumor characteristics is increasingly important. Future audits should include structured MDT decision fields, treatment intent, reasons for nonoperative management, and reasons for incomplete chemotherapy to better understand treatment pathways.

Real-world vs. clinical trial outcomes

This study highlights the gap between clinical trial outcomes and real-world practice. Clinical trials often include highly selected patients, whereas real-world cohorts reflect broader patient populations with greater complexity [10].

Strengths

The strengths of this study include the use of real-world data from an NHS setting, which enhances the generalizability of the findings to routine clinical practice; a long study duration of five years, allowing for meaningful outcome assessment over time; and a comprehensive evaluation of clinical outcomes, including treatment patterns, complications, and descriptive PFS in a cohort receiving active treatment.

Limitations

This study has several limitations. It was a retrospective single-health-board audit with a small sample size and an active-treatment cohort, limiting generalizability to all patients with UGI cancer. Multiple variables were incompletely or inconsistently documented, including ECOG performance status, comorbidity burden, overall clinical stage grouping, treatment intent, dose reduction, surgical procedure type, structured complication grading, median follow-up, event timing, and censoring status. Patient-level overall postoperative complication incidence could not be reliably calculated. Kaplan-Meier analysis, median PFS, median follow-up, confidence intervals, and inferential statistical testing were not performed. These limitations mean that findings should be regarded as descriptive.

Future directions

Future work should focus on prospective data collection using a predefined audit proforma, standardized definitions of postoperative complications, Clavien-Dindo and CTCAE grading, accurate chemotherapy completion and dose-modification data, structured follow-up dates, recurrence/progression dates, and time-to-event analysis. Multicenter collaboration may improve sample size and generalizability. Prospective evaluation would also allow more rigorous assessment of perioperative optimization, prehabilitation, enhanced recovery pathways, and MDT processes.

## Conclusions

UGI cancers continue to pose a significant clinical challenge due to aggressive biology and frequent late-stage presentation. This retrospective institutional audit provides descriptive real-world data on treatment patterns and outcomes for 78 patients with UGI cancers managed within an NHS setting in North Wales. Among 78 patients receiving active treatment, all received neoadjuvant FLOT chemotherapy, and 56 underwent or had attempted surgical resection. Of 78 patients, crude-documented two-year progression-free status was observed in 30 patients (38.5%), 32 (41.0%) had documented progression or death within two years, and 16 (20.5%) were not evaluable or not recorded. Thirty-day postoperative mortality among surgically treated patients was one of 56 (1.8%). Documented postoperative events included sepsis, pneumonia, and anastomotic leak, but reliable patient-level overall complication incidence and formal complication grading were not available.

These findings highlight the challenges of managing advanced UGI cancer in routine clinical practice and support continued evaluation of multimodal treatment approaches. The findings should be interpreted as descriptive real-world audit data rather than formal survival analysis or evidence of equivalence with clinical trial outcomes. While optimizing perioperative care, implementing prehabilitation/ERAS protocols, and strengthening multidisciplinary coordination are important strategies that may improve outcomes, the present retrospective descriptive data do not permit direct causal inference regarding the impact of these interventions in this specific cohort. Future prospective audits with standardized baseline, treatment, complication, and follow-up data are needed to better characterize outcomes and evaluate perioperative service-improvement interventions in this population.
